# Evaluation of Biofeedback Usefulness in Masticatory Muscle Activity Management—A Systematic Review

**DOI:** 10.3390/jcm8060766

**Published:** 2019-05-30

**Authors:** Wojciech Florjanski, Andrzej Malysa, Sylwia Orzeszek, Joanna Smardz, Anna Olchowy, Anna Paradowska-Stolarz, Mieszko Wieckiewicz

**Affiliations:** 1 Department of Experimental Dentistry, Faculty of Dentistry, Wroclaw Medical University, 50-425 Wroclaw, Poland; wojtek.florjanski@gmail.com (W.F.); andrzejmalysa@o2.pl (A.M.); sylwia.winiewska87@gmail.com (S.O.); joannasmardz1@gmail.com (J.S.); ania.olchowy@gmail.com (A.O.); 2 Division of Facial Abnormalities, Department of Maxillofacial Orthopaedics and Orthodontics, Faculty of Dentistry, Wroclaw Medical University, 50-425 Wroclaw, Poland; annamaria.paradowska@gmail.com

**Keywords:** biofeedback, masticatory muscle activity, masseter muscle activity, temporalis muscle activity, temporomandibular disorders

## Abstract

Temporomandibular disorders (TMD) have multifactorial and complex etiology. Regardless of their etiology, all those conditions may result in centrally mediated chronic muscle pain, myalgia, myofascial pain, myofibrotic contracture, myosistis, myospasm, headache and a variety of neck, shoulder, upper back and lower back pain. Biofeedback (BF) is one of methods that has been used for more than 50 years in rehabilitation to facilitate normal movement patterns after injuries. Some studies suggest that biofeedback may be an effective treatment option for patients with different muscle disorders. The aim of this study was to evaluate the efficiency of biofeedback in masticatory muscle activity management in the light of current medical literature. The authors followed the Preferred Reporting Items for Systematic Review and Meta-Analysis (PRISMA) guidelines for this systematic review. The authors searched the MEDLINE, Scopus, Web of Science, CINAHL databases to identify relevant publications. Finally 10 papers were included. Most of the selected studies showed a significant correlation between biofeedback usage and reduction of masticatory muscle activity. By analyzing qualified studies, it can be concluded that biofeedback can be an effective tool in masticatory muscle activity management.

## 1. Introduction

Temporomandibular disorders (TMD) have multifactorial and complex etiology. The current biopsychosocial model includes such components as the anatomy of the temporomandibular joint (TMJ), psychological and emotional factors, and systematic disorders such as fibromyalgia or neuromuscular changes [1,2]. Also such behaviors as sleep bruxism (SB) or awake bruxism (AB) might be considered as risk factors of temporomandibular disorders [3].

Regardless of the etiology, all those conditions may result in centrally mediated chronic muscle pain, myalgia, myofascial pain, myofibrotic contracture, myosistis, myospasm, headaches and a variety of neck, shoulder, upper back and lower back pain. Other than pain, TMD are characterized by difficulty in maximal opening of the jaw, locking in the open or closed position, and clicking or grating sounds in TMJ [2,4]. 

Current literature has suggested that temporomandibular disorders patients may suffer from dysfunction in the brain network that supports sensory, pain, emotional, and cognitive processes [5,6]. Some authors have focused on the dysregulation of the autonomic nervous system (ANS) in TMD patients [7,8], suggesting that TMD could be the clinical manifestation of multisystemic dysregulation. 

In order to regulate muscle tension and decrease muscle pain, various techniques have been used with different results [9]. 

Biofeedback (BF) is one of those methods. It has been used for more than 50 years in rehabilitation to facilitate normal movement patterns after injuries [10]. It is the technique of providing biological information to patients in real-time that would otherwise be unknown. This information can sometimes be referred to as augmented or extrinsic feedback—that is, feedback that provides the user with additional information, above and beyond the information that is naturally available to them as opposed to the sensory (or intrinsic) feedback that provides self-generated information to the user from various intrinsic sensory receptors [11].

To provide feedback from muscles, electromyography biofeedback (EMG-biofeedback) is used. In this method, surface electrodes are placed on the skin to measure frequency, intensity and duration of muscle contraction [12]. It can be used to either increase activity in weak or paretic muscle or to facilitate a reduction in tone of a spastic one. EMG biofeedback has been shown to be useful in both musculoskeletal and neurological rehabilitation [13].

Two types of protocols are in use: biofeedback training (BFB training) and contingent electrical stimulation (CES).

BFB training is a method, in which a vigil patient modifies muscle activity with self-control, based on a constant feedback of a registered signal [14].

CES is a method, which does not require conscious activity. The device emits a non-painful electrical pulse to the chosen muscle region when EMG activity exceeds the individually determined threshold [15].

Due to the absence of agreement about an effective unified management for temporomandibular disorders, non-invasive therapies such as biofeedback generate greater interest. Furthermore, most studies to the present show methodological deficiencies that must be solved in the future, which makes it important to emphasize this line of studies. 

EMG-biofeedback is the most widely used and reported method of biofeedback. However, the limited number of large randomized controlled trials and systematic reviews means further work is required. Existing evidence for the use of biofeedback in musculoskeletal and neurological rehabilitation appears promising.

The purpose of this study is to review the available modern literature to determine the effects and efficiency of masticatory muscle activity management based on biofeedback. In the opinion of the authors, the creation of such a systematic review was necessary due to the great progress in work on the use of biofeedback in the last 20 years and its unspecified contribution to masticatory muscle activity management.

## 2. Material and Methods

The authors of this paper followed the Preferred Reporting Items for Systematic Review and Meta-Analysis (PRISMA) guidelines for our systematic review and to collect and report data [16,17].

### 2.1. Studies

The authors established the following inclusion criteria: randomized controlled trials, prospective and retrospective cohort studies that discussed biofeedback usage in TMD. English language and full text peer-reviewed articles published between January 1998 and January 2019 were included in the study. 

### 2.2. Participants

Participants were males and females of any age with clinical diagnosis of TMD treated with biofeedback in any protocol.

### 2.3. Outcomes

A study was included into this systematic review if it investigated the primary outcomes of interest: efficiency of biofeedback in management of masticatory muscle activity such as influence on: muscle activity, pain and bruxism episodes.

### 2.4. Data Sources and Searches

The authors searched the MEDLINE, Scopus, Web of Science, CINAHL databases to identify relevant publications. The authors added filters to identify randomized controlled trials, prospective and retrospective cohort studies to ensure the searching process were accurate. Moreover filters included articles published between January 1998 and January 2019 and those available in English. The literature search strategy was based on medical subject headings (MeSH) [14,15] as follows: each of three synonymous phrases, i.e., (1) EMG-biofeedback, (2) Biofeedback, (3) EMG biofeedback were combined with each of: (a) TMD, (b) temporomandibular disorders, (c) temporomandibular disorder, (d) masticatory muscle, (e) masticatory muscles. Example: “EMG-biofeedback TMD,” viz. (1) + (a); “EMG-biofeedback temporomandibular disorders”, viz. (1) + (b), etc. In this way, 15 queries were obtained. The reference list of included studies was also screened to identify other potentially appropriate studies.

### 2.5. Trial Selection

Four authors (W.F., S.O., A.M. and A.O.) searched literature for potentially relevant articles. Firstly, the titles and abstracts were screened for key phrases, such as “biofeedback”, “EMG-biofeedback”, “EMG biofeedback”, “temporomandibular disorders”, “TMD”, “masticatory muscle”, “masticatory muscle disorders”, “masticatory muscle pain”, “masticatory myofascial pain”, “masticatory muscle spasm”, “masseter”, “temporalis” independently by all four authors. Next, based on abstracts, potentially suitable manuscripts were selected for assessment. In the next step the full texts of the chosen articles were evaluated in the context of the research question. Finally, the authors decided together if all of the chosen articles fulfilled the inclusion criteria. Disagreements were resolved by consensus. None of the review authors was blind to the journal title or to the study authors or institutions [17].

### 2.6. Data Extraction

After the final agreed decision, three reviewers conducted data extraction independently (W.F., A.M., S.O). Then, the fourth author (M.W.) checked the validity of all data extracted.

The data extraction process focused on the information about sample size and gender, primary diagnosis, diagnostic criteria, type of biofeedback intervention, number of treatment sessions, time of each session main outcomes, and tools used to measure those outcomes.

### 2.7. Data Synthesis and Analysis

The authors conducted a narrative, qualitative summary. The Grading of Recommendations Assessment, Development and Evaluation working group approach was used to assess the quality of evidence. Included studies were evaluated in accordance to factors and criteria such as studied problem, values and preferences, quality of evidence, benefits and harms and burden, resource implications, equity, acceptability, feasibility. One of the following categories: very low (the true effect is likely to be substantially different from the estimate of effect), low (the true effect may be substantially different from the estimate of the effect), moderate (the true effect is likely to be close to the estimate of the effect, but there is a possibility that it is substantially different), or high (the true effect lies close to that of the estimate of the effect), was used to assess the quality of evidence for outcome [15].

**Figure 1:**
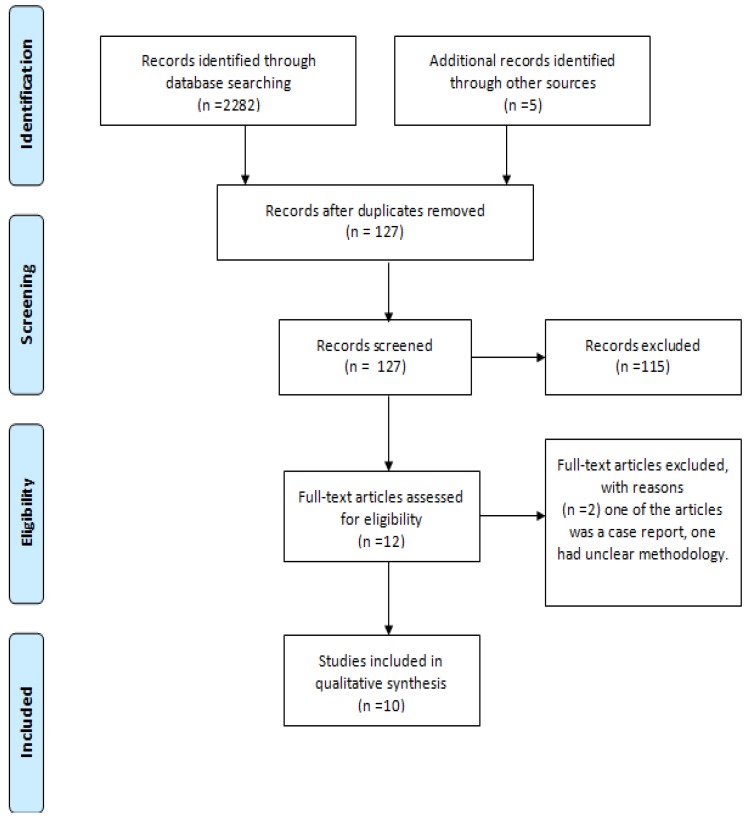
Flow diagram of the systematic review protocol.

The protocol of the systematic review is presented as a flow diagram in Figure 1. 

## 3. Results

The authors obtained 2282 results from January 1998 to January 2019 in accordance with the research protocol. After duplicates removal, only 127 articles were left. After screening for key words and abstract reading, 115 papers were excluded and 12 remaining articles were assessed for eligibility. Finally only 10 studies were included. One paper was excluded due to unclear methodology and the other one was a case report which presented low quality of evidence. 

### 3.1. Characteristics of the Subjects Included in the Primary Studies

The total number of participants included in the individual studies ranged from 10 to 24. Subjects were adults, mainly females. Only in one study were participants male only [18]. The age of patients ranged from 20 years to 60 years. Participants suffered from TMD-related muscle pain [14,19,20], myofascial pain [15,21] sleep bruxism [15,18,21,22,23,24,25], awake bruxism [14,18,19] and in one case the type of bruxism was not defined [20].

### 3.2. Quality Assessment

The authors finally included 10 papers—crossover studies, single-blinded, randomized clinical trials. They also reported this kind of study to be the most reliable. The studies were divided into two groups, depending on the type of biofeedback intervention used: biofeedback training and contingent electrical stimulation. In biofeedback training patients received signals such as: audio signals [18,19,22], visual signals [14,20], and vibratory signals [24] encouraging them to perform certain actions. The majority of the included studies used EMG electrodes as the receiver of changes in participants’ organisms [14,15,18,19,20,21,22,23]; in one case electrocardiogram electrodes were used [25] and in one case pressure sensors embedded in a maxillary splint were used [24]. EMG electrodes were placed over the masseter muscle [22,25] or temporalis muscle [15,18,19,21,23], or both [14,20]. Only research investigating awake bruxism explained the position of electrodes. In those studies the temporalis muscle was chosen, because the electrode unit was less noticeable, especially when covered by hair, and did not disturb subjects normal day activities [18,19]. The number of BFB-training sessions varied from 2 [19] up to 4 [14,18], in one case the number was not given [24]. Each session lasted from 20 min [20] up to 8 h [22]. In CES studies, the treatment lasted from 2 nights [25] up to each night of 6 weeks [21]. The included studies present a huge variety of study designs, protocols of biofeedback treatment and signals on which the biofeedback is based. 

Information about the type of intervention, electrode placement, sample size, biofeedback group sample, diagnosis, diagnostic criteria, outcomes, tools used to measure outcomes, number of sessions or time of active treatment and time of each session are presented in Table 1 and Table 2.

### 3.3. Synthesis of Evidence

Table 3 presents the quality of the evidence as an overall grade score for the primary outcome. The initial grade score of included studies was decreased due to the study design. Other common causes of reduced scores were clinical heterogeneity between studies and indirectness.

### 3.4. Primary Outcome

Most of the studies suggested a significant correlation between usage of biofeedback and reduction of masticatory muscle activity [14,15,18,19,21,23,24,25], only in one case the tendency was marked but not statistically significant [20]. 

The quality of evidence in this group of studies was in 3 cases very low, in 6 cases low, and in 1 case moderate. 

## 4. Discussion

The greatest strength of the presented systematic review is the methodology of studies search. This systematic review presents research over the past 21 years, which is a key period in the development of research on the effectiveness of biofeedback. Additionally, the proposed search system allowed an accurate and multi-level search of many scientific databases for relevant publications. Like any research, it also has limitations. Authors could finally include only 10 studies and in most of them sample sizes were small, which could potentially affect the clinical quality of the research included. The approach to TMD has changed over the last few decades from occlusion focused models to biopsychosocial model. 

The majority of included studies presented a significant correlation between biofeedback usage and reduction of muscle activity [14,15,18,19,21,22,23,24,25]. However, the quality of evidence of included studies is debatable. The number of participants ranged from 10 [22,25] to 24 [24], in two articles due to randomized control trial protocol usage, the active biofeedback group was limited to 7 patients [15,18]. The studies varied from short-term studies lasting a few days [19,22,25] up to studies lasting a few weeks [14,15,18,20,21,23,24]. Only two of the studies had a follow-up [15,21]. In research conducted by Conti et al. right after the CES treatment, participants ware an inactive portable EMG device for at least 5 nights. The reduction of EMG events per hour of sleep persisted, but in comparison to the control group the result was not statistically significant anymore [15]. In the study conducted by Raphael et al. significant reduction of muscle activity pre-post trial was found, but the follow-up (after 2 weeks) showed that EMG events per minute of sleep returned to the levels from the beginning of the trial [21]. 

Some of the included studies were concerned with perceived pain [14,15,21] and sleep quality [22,23,24]. 

In some studies pain-related symptoms were discussed [14,15,21,23]. A wide variety of symptoms analyzed by each author make comparison of obtained data extremely difficult and the impact of biofeedback on pain unclear. In the study conducted by Criado et al. perceived pain symptoms such as muscle pain and pain during jaw movement did improve after the first session of biofeedback. All participants registered significant decrease of symptoms such as: muscle pain, pain at the opening movement, pain during and radiated pain after the last session of biofeedback in comparison to previous ones [14]. Raphael et al. reported reduction of pain during palpation and self-reported pain (current pain intensity) after CES treatment and also that these effects persisted during follow-up (2 weeks after the end of the trial). However, correlation between the change in the number of EMG events (the direct effect of biofeedback treatment used) and both subjective and objective pain symptoms was described as doubtful [21]. On the other hand, in the study conducted by Conti et al. such parameters as: present pain intensity and pressure pain threshold did not change in comparison to baseline levels after conducted CES treatment. In addition the authors noted no correlation between the reduction of EMG events per hour and the above mentioned parameters [15]. Jadidi et al. examined number of painful muscles, characteristic pain intensity and maximum pain-free jaw opening and found no statistically significant changes post-trial in comparison to baseline values [23].

Some trials concerned with sleep bruxism also investigated the influence of biofeedback interventions on sleep parameters [22,23,24]. All suggested that biofeedback does not have a negative impact on sleep. Some based their opinion just on the overall amount of time the patient slept during the study period [23], some conducted EEG sleep stages evaluation [22], some measured biochemical parameters indicating physical stress after sleep (chromogramin A in saliva) [22], and some based on self-reported data using questionnaires such as: Pittsburgh Sleep Quality Index (PSQI) [24] or State Trait Anxiety Inventory [22]. Also, a study focusing on polysomnographic parameters in patients with sleep bruxism undergoing CES therapy confirmed that the CES treatment does not affect the total time of sleep, number of micro-arousals and stages of sleep [26].

In none of the discussed studies morphological changes in masticatory muscle were investigated, although such changes as muscle thickening, muscle hardening or muscle edema might be observed in TMD patients [27,28]. Also, occlusal factors were not discussed in any of presented studies probably due to their diminishing role in TMD etiology [29,30]. After all, it is worth mentioning that masticatory muscles are involved in a broad range of different activities such as chewing, sucking, swallowing and speech. It determines their unique fibers composition structure. Myosin protein isoforms production in human masticatory muscles can be changed by genetic and environmental factors. This can modulate genetic variation of muscle fibers and their adaptive response [31]. This can be important when taking into account the different response of each individual to the same biofeedback treatment protocol. Another important factor that can modulate the therapeutic effectiveness of biofeedback is chronic stress. Schmitter et al. in a pilot study on females concerning chronic stress and temporalis muscle activity in TMD patients and controls during sleep concluded that work-related chronic stress seemed to be associated with an increased level of temporalis muscle activity during sleep [32]. Therefore, if stress can affect the activity of masticatory muscles, its elimination could increase the therapeutic effectiveness of biofeedback.

## 5. Conclusions

By analyzing qualified studies, it can be concluded that biofeedback is useful in decreasing masticatory muscle activity. However, further studies on a larger group of participants taking into account coexisting genetic and environmental factors that can modify the effect of biofeedback on masticatory muscles are needed to verify the results of the treatment and long-term follow-ups in order to clarify permanence. Also, the efficiency of different protocols remains unclear.

## Figures and Tables

**Table 1:** Summary of included studies using biofeedback training.

Authors	Type of Intervention	Electrode Placement	Sample Size	BFB Group Sample	Diagnose	Diagnostic Criteria	Outcomes	Tools Used to Measure Outcomes	Number of Sessions/Time of Active Treatment	Time of Each Session
1. Wieselmann-Penker et al. (2001) [20]	Visual BFB training vs. TENS treatment	Masseter, temporalis	*N* = 20 (13 f, 7 m)	10	Bruxism (type not defined), Muscle TMD	No data	Marked tendency of reduction of mean EMG level after BFB and TENS	EMG analysis (pre-, post- training)	3 (1 per week)	20 min. 10 min. for masseter and 10 for temporalis
2.Gu et al. (2015) [24]	Vibratory BFB vs. occlusal splint	No electrodes, pressure sensor in the device	*N* = 24 (19 f, 5 m)	*N* = 12 (9 f, 3 m)	SB	Criteria by AASM	Number and time of SB events significantly decreased	Number and time of SB events pre-, post- trial)	12 week therapy	No precise data
3.Criado et al. (2016) [14]	Visual BFB training	Masseter temporalis	*N* = 14 (7 f, 7 m)	*N* = 14 (7 f, 7 m)	Muscle TMD AB	RDC/TMD self-report	Decrease of pain perceived, decrease of EMG-muscle activity	Clinical evaluation, questionnaires, NRS, EMG analysis	4 (2 times a week for 2 weeks)	No precise data (30 iterations)
4.Watanabe et al. (2011) [19]	Audio BFB vs. CO	Temporalis	*N* = 20	*N* = 10	Muscle TMD, AB	Self-report	Decrease of daytime clenching events in BFB group	EMG analysis pre-post- trial	2 (2 consecutive days)	5 h
5.Sato et al. (2015) [18]	Audio BFB vs. CO	Temporalis	*N* = 12 (12 m)	*N* = 7 (7 m)	AB and SB	Self-report	Decrease of tonic events number for both SB and AB	EMG analysis	4 (2 consecutive days—2 day and 2 night sessions)	5 h
6.Goto et al. (2015) [22]	Audio BFB	Masseter	*N* = 10 (5 f, 5 m)	*N* = 10 (5 f, 5 m)	SB	No data	Decrease of SB events	EMG analysis	3 (3 consecutive nights)	8 h

BFB: biofeedback; TENS: transcutaneous electrical neuromuscular stimulation; m: male; f: female; TMD: temporomandibular disorders; EMG: electromyography; SB: sleep bruxism; AB: awake bruxism; AASM: American Academy of Sleep Medicine; NRS: numeral rating scale; RDC/TMD: Research Diagnostic Criteria for Temporomandibular Disorders; CO: control group.

**Table 2:** Summary of included studies using contingent electrical stimulation.

Authors	Type of Intervention	Electrode Placement	Sample Size	BFB Group Sample	Diagnose	Diagnose Criteria	Outcomes	Tools Used to Measure Outcomes	Nr of Sessions/Time of Active Treatment
1.Conti et al. (2014) [15]	CES vs. CO	Temporalis	*N* = 15 (12 f, 3 m)	*N* = 7 (5 f, 2 m)	Myofascial pain, SB	RDC/TMD AASM	Significant reduction EMG events per hour of sleep, no changes in present pain intensity and pressure pain threshold	VAS, algometry, EMG analysis	At least 10 days
2.Raphael et al. (2013) [21]	CES	Temporalis	*N* = 14 (14 f)	*N* = 14 (14 f)	Myofascial pain, SB	RDC/TMD PSG	Significant reduction of EMG activity during treatment, with return to base line in follow-up. No changes in self-reported night pain Significant reduction of perceived pain after palpation and spontaneous, also in follow-up	EMG analysis (EMG events per min. of sleep), NRS, RDC/TMD	Each night for 6 weeks
3.Jadidi et al. (2008) [23]	CES	Temporalis	*N* = 14 (8 f, 6 m)	*N* = 14 (8 f, 6 m)	SB	AASM	Significant reduction of EMG events/hour of sleep in active CES phase of the study and in inactive phase of the study No changes in perceived pain	EMG analysis (number of EMG events/h of sleep), RDC/TMD	5–7 nights a week for 6 weeks (3 weeks with a 2-week break and another 3 weeks)
4.Sumiya et al. (2014) [25]	CES	Masseter	*N* = 10 (4 f, 6 m)	*N* = 10 (4 f, 6 m)	SB	EMG monitoring by night	Significant decrease of EMG events/h of sleep and events/night, Significant decrease of number of burst and duration of SB	EMG analysis (events/h of sleep and events/night, number of burst of SB event, duration of SB events)	2 consecutive nights

CES: contingent electrical stimulation; CO: control group; VAS: visual analog scale; EMG: electromyography; f: female; m: male; SB: sleep bruxism; RDC/TMD: Research Diagnostic Criteria for Temporomandibular Disorders; AASM: American Academy of Sleep Medicine; PSG: polysomnography; NRS: numeral rating scale.

**Table 3:** Summary findings for the primary outcome.

No	Outcome Significance	Trials (Year)	Quality of the Evidence (Grade)
1	No significant correlation	Wieselmann-Penker et al. (2001) [20]	+ + − − low due to indirectness, imprecision
2	Significant correlation	Conti et al. (2014) [15]	+ + − − low due to indirectness, imprecision
3		Gu et al. (2015) [24]	+ + + − moderate due to indirectness
4		Sato et al. (2015) [18]	+ + − − low due to indirectness, imprecision
5		Criado et al. (2016) [14]	+ + − − low due to indirectness, imprecision
6		Watanabe et al. (2011) [19]	+ − − − very low due to indirectness, imprecision, inconsistency
7		Goto et al. (2015) [22]	+ − − − very low due to indirectness, imprecision, inconsistency
8		Jadidi et al. (2008) [23]	+ + − − low due to indirectness, imprecision
9		Raphael et al. (2013) [21]	+ + − − low due to indirectness, imprecision
10		Sumiya et al. (2014) [25]	+ − − −very low due to imprecision, indirections, inconsistency

Quality of evidence: ++++ high, +++− moderate, ++−−low, +−−− very low.
